# Gut Microbiota‐Derived Metabolites Regulate CASP3 and Neuroimmune Pathways in Multiple Sclerosis: An Integrative Multiomics Study

**DOI:** 10.1155/mi/3572399

**Published:** 2026-02-10

**Authors:** Li Li, Hongwei Liu, Zhinan Ye, Wenbin Xu

**Affiliations:** ^1^ Department of Neurology, Shanxi Provincial People’s Hospital, Taiyuan, Shanxi Province, China, spph-sx.com; ^2^ Department of Neurology, Xuanwu Hospital of Capital Medical University, Beijing, China, xwhosp.com.cn; ^3^ Department of Neurology, Taiyuan City Central Hospital, The Ninth Clinical Medical College of Shanxi Medical University, Taiyuan, Shanxi Province, China; ^4^ Department of Neurology, Taizhou Municipal Hospital (Taizhou University Affiliated Municipal Hospital), School of Medicine, Taizhou University, Taizhou, Zhejiang Province, China, tzc.edu.cn; ^5^ Department of Rehabilitation Medicine, Taizhou Municipal Hospital (Taizhou University Affiliated Municipal Hospital), School of Medicine, Taizhou University, Taizhou, Zhejiang Province, China, tzc.edu.cn

**Keywords:** CASP3, gut microbiota, immune regulation, metabolites, multiple sclerosis

## Abstract

**Background:**

Multiple sclerosis (MS) is a chronic autoimmune disease of the central nervous system, closely associated with neuroinflammation, immune dysregulation, and gut microbiota imbalance. Gut microbiota‐derived metabolites may modulate key targets involved in MS pathogenesis.

**Methods:**

This study integrated network pharmacology, machine learning (ML), and single‐cell transcriptome analysis to identify MS‐related differentially expressed genes (DEGs) and potential targets of gut microbial metabolites. Feature contributions were evaluated using the SHapley Additive exPlanations (SHAP) method, and causal relationships were validated via Mendelian randomization (MR). Single‐cell analysis, molecular docking, and assessments of drug‐likeness and toxicity were also performed.

**Results:**

Caspase‐3 (CASP3) was identified as a core target interacting with multiple gut microbial metabolites, including L‐isoleucine, aromatic lactic acid derivatives, 3‐hydroxyphenethyl alcohol, and D‐xylose, potentially regulating neuroimmune responses via TNF, MAPK, IL‐17, and galectin pathways. Specific microbial taxa, such as *Akkermansia*, *Bacteroides*, and *Bifidobacterium*, were closely associated with these metabolites. The metabolites exhibited favorable drug‐likeness and low predicted toxicity, indicating potential therapeutic value.

**Conclusion:**

Gut microbial dysbiosis and its metabolites play a significant role in MS onset and progression, providing a theoretical basis for identifying therapeutic targets and gut–CNS axis interventions. Experimental validation is needed to confirm mechanisms and translational potential.

## 1. Introduction

Multiple sclerosis (MS) is a chronic inflammatory autoimmune disease primarily affecting the central nervous system, characterized by inflammatory demyelinating lesions in the white matter, axonal injury, and neurological dysfunction [1, 2]. MS is one of the leading causes of neurological disability in young adults, typically occurring between the ages of 20 and 40 [3]. Pathologically, MS is manifested by demyelination, axonal damage, and glial cell proliferation, predominantly affecting both white and gray matter regions of the brain and spinal cord [4]. Globally, approximately 2.8 million people live with MS, with a median incidence of 2.1 cases per 100,000 population per year and a mean age at diagnosis of 32 years [5]. The etiology of MS remains incompletely understood, but studies suggest that its onset is likely associated with multiple factors, including autoimmunity, genetic susceptibility, environmental influences, and microbial infections [6–8]. Notably, MS exhibits a pronounced sex bias, with women affected approximately two to three times more frequently than men. This phenomenon has been consistently observed across different populations, suggesting that sex‐specific genetic, hormonal, and immunological factors may play an important role in MS susceptibility [9–11].

In recent years, growing evidence has indicated a close relationship between MS and the gut microbiota [12, 13]. The composition of the gut microbiota in MS patients differs significantly from that of healthy individuals in both diversity and abundance. For example, the abundance of *Faecalibacterium* is reduced, whereas *Prevotella* is increased in the gut of MS patients, and these alterations may be associated with disease onset and progression [14]. Furthermore, dysbiosis of the gut microbiota may influence immune responses and neuroinflammatory processes in MS [15–17]. Studies have shown that the population of short‐chain fatty acid (SCFA)‐producing bacteria is reduced in the gut of MS patients, which may lead to abnormal immune activation and disruption of the blood–brain barrier [18]. These findings suggest that the gut microbiota may contribute to the development and progression of MS by modulating the immune system and neuroinflammation, providing new insights for early diagnosis and therapeutic strategies.

Recent advances in high‐throughput technologies have improved our understanding of cellular communication. Mendelian randomization (MR) leverages genetic variants to infer potential causal relationships [19]. Machine learning (ML) detects hidden patterns in large datasets, and frameworks like SHapley Additive exPlanations (SHAP) help interpret ML predictions by quantifying feature contributions [20, 21]. Currently, these approaches are widely used to elucidate disease mechanisms and identify potential therapeutic targets.

**Figure 1:**
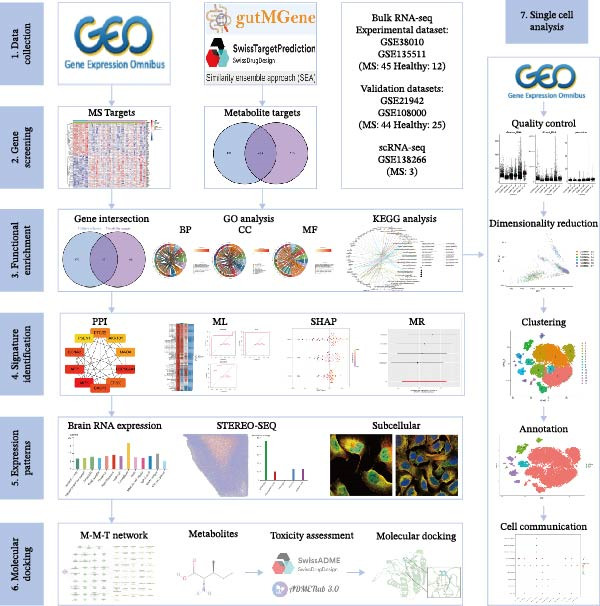
Flowchart of this study.

This study integrates network pharmacology, ML, and single‐cell transcriptome analysis to systematically investigate the regulatory effects of gut microbial metabolites on key targets in MS. We first identified core targets using ML combined with the game‐theory‐based SHAP method and validated their potential causal relationships via MR. Subsequently, the differentially expressed genes (DEGs) of these targets and their enriched pathways were analyzed at the single‐cell level. Finally, molecular docking was performed to evaluate the interactions between gut microbiota‐derived metabolites and core targets (Figure 1). This integrative strategy may provide new theoretical insights and potential therapeutic targets for understanding MS pathogenesis and gut microbiota‐related interventions, laying a solid foundation for future experimental studies.

## 2. Methodology

### 2.1. Data Source

Figure 1 is the overall workflow of this study. In this study, disease‐related datasets (GSE38010 and GSE135511) were retrieved from the GEO database (https://www.ncbi.nlm.nih.gov/geo/), and data preprocessing was performed using R software (version 4.3.1). DEGs were defined with the criteria of |log_2_ fold change| ≥ 1 and an adjusted *p* ≤ 0.05 (Benjamini–Hochberg correction). The resulting DEGs were visualized using heatmaps generated with the “pheatmap” and “ggplot2” packages. Additionally, GSE21942 and GSE108000 were used as validation datasets.

### 2.2. Identification of Metabolites and Targets From Gut Microbiota

The metabolites of the gut microbiota were obtained from the gutMGene database (http://bio-annotation.cn/gutmgene/). The SMILES structures of these metabolites were submitted separately to the Similarity Ensemble Approach (SEA, https://sea.bkslab.org/) and Swiss Target Prediction (STP, http://swisstargetprediction.ch/) to identify potential targets. The overlapping targets between SEA and STP were considered the core targets of the gut microbiota metabolites.

### 2.3. Functional Enrichment Analysis

Gene Ontology (GO) annotation and Kyoto Encyclopedia of Genes and Genomes (KEGG) pathway enrichment analyses were conducted using the “clusterProfiler” R package (V. 4.8.3) to explore the biological roles and signaling pathways linked to the DEGs. False discovery rate (FDR) threshold < 0.05, *p*  < 0.05.

### 2.4. ML

To identify potential biomarkers for MS, twelve ML algorithms, including RF, SVM, Lasso, XGBoost, Enet, GBM, Ridge, LDA, glmBoost, Stepglm, Naïve Bayes, and plsRglm, were combined into 110 distinct model configurations to construct diagnostic models. Model performance was primarily evaluated using the concordance index (C‐index), and the top‐performing model was selected for downstream analysis. Predictive accuracy was further quantified by calculating the area under the ROC curve (AUC) with the “timeROC” R package.

### 2.5. SHAP Analysis

Based on the selected predictive factors, we constructed ten ML models (random forest, support vector machine, logistic regression, ridge least squares, decision tree, k‐nearest neighbors, gradient boosting machine, extreme gradient boosting, neural network, and generalized linear model boosting), including both highly interpretable models suitable for analyzing linear relationships and models capable of capturing nonlinear relationships and variable interactions, suitable for handling high‐dimensional data. The final model was interpreted using the SHAP method to evaluate the contribution of each feature to the predictions, providing insights into the underlying mechanisms.

### 2.6. MR Analysis

We performed MR analysis using the inverse variance weighted (IVW) method as the primary estimator. Single nucleotide polymorphisms (SNPs) significantly associated with the exposure (*p* < 5 × 10^−6^) were pruned for linkage disequilibrium (*R* ^2^ < 0.001, distance > 10,000 kb) to ensure independence. Instrument strength was assessed by calculating F‐statistics, with all values > 10, indicating no weak instrument bias. Horizontal pleiotropy was evaluated using the MR–Egger intercept test (*p* > 0.05) and further examined by MR‐PRESSO to detect and remove potential outliers.

### 2.7. Single‐Cell Transcriptomic Profiling

Single‐cell RNA sequencing (scRNA‐seq) datasets were subjected to quality control by excluding cells that expressed fewer than 500 genes or exhibited more than 25% mitochondrial gene content. Additionally, contaminating red blood cells and ribosomal transcripts were removed to ensure high‐quality data. Following this, the expression values were log‐normalized and batch effects across samples were corrected using the IntegrateData function in Seurat. The resulting dataset was scaled with the ScaleData function to standardize expression levels. Cell identities were inferred through SingleR annotation, leveraging reference transcriptomes. For visualization and dimensionality reduction, t‐distributed stochastic neighbor embedding (tSNE) was applied. Clustering of cells was performed using Seurat’s FindClusters algorithm, and each cluster was annotated based on canonical marker genes to assign biological cell types. This workflow allowed for a robust characterization of cellular heterogeneity within the analyzed samples. Finally, CellChat (V.1.6.1) was employed to analyze intercellular communication in cerebrospinal fluid (CSF) samples from MS patients.

### 2.8. Molecular Docking

The caspase‐3 (CASP3) protein structure (AF‐P42574‐F1‐v4) was obtained from the AlphaFold database (https://alphafold.ebi.ac.uk/). Structures of gut microbiota‐related metabolites were retrieved from the PubChem database (https://pubchem.ncbi.nlm.nih.gov/). Molecular docking between CASP3 and the metabolites was performed using the CB‐Dock2 platform (https://cadd.labshare.cn/cb-dock2/php/blinddock.php#job_list_load), and binding energies were recorded. Finally, docking results were visualized using PyMOL.

## 3. Results

### 3.1. Screening of DEGs and Metabolite Targets

After integrating the GSE38010 and GSE135511 datasets, a total of 1427 DEGs were identified (Figure 2A). DEGs were selected using the criteria of |log_2_ fold change| ≥ 1 and an adjusted *p* ≤ 0.05. Subsequently, 668 potential targets of gut microbiota metabolites were predicted using the SEA and STP databases (Figure 2B), and intersection with MS‐related DEGs yielded fifty seven candidate genes (Figure 2C,G). These genes may be associated with MS pathogenesis, and we will continue to screen them in subsequent studies.

Figure 2Differential expression analysis and functional enrichment analysis. (A) Heatmap of DEGs, (B) targets of gut microbiota metabolites, (C) common regulatory targets of the gut microbiota and MS, GO enrichment analysis, including BP (D), CC (E), and MF (F), (G) network construction of gut metabolites and MS targets, and (H) KEGG pathway enrichment analysis.

**Figure:**
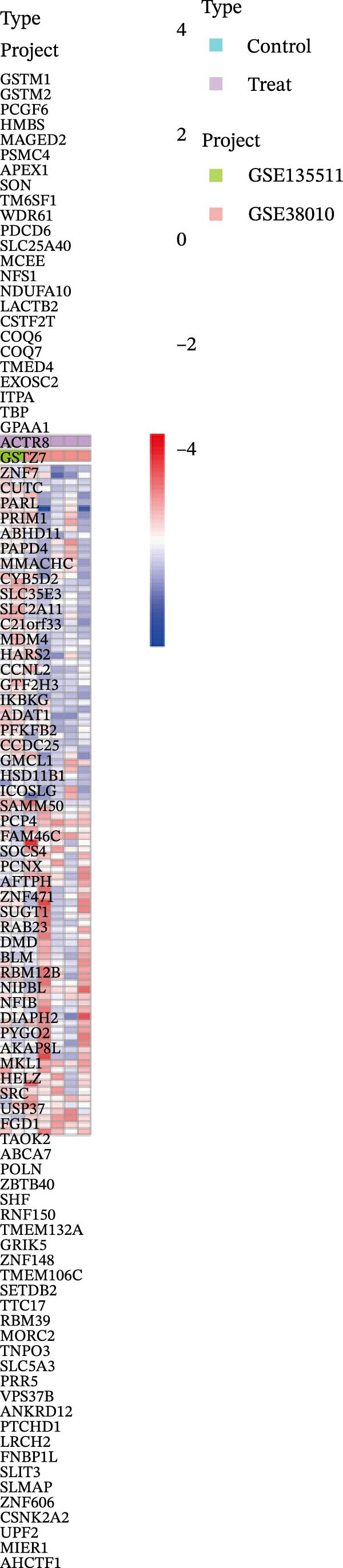
(A)

**Figure:**
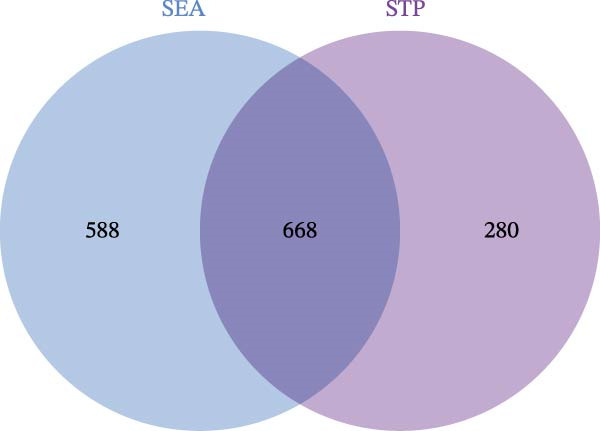
(B)

**Figure:**
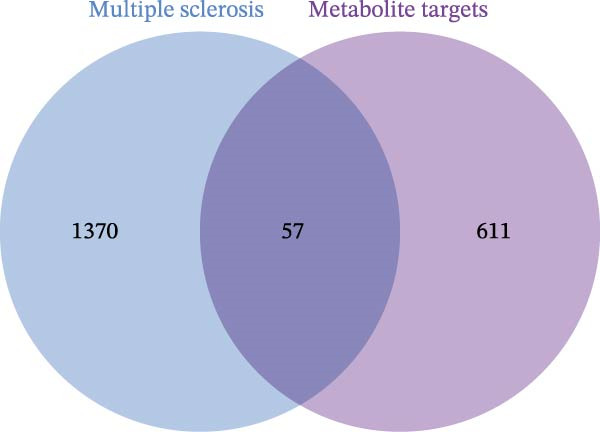
(C)

**Figure:**
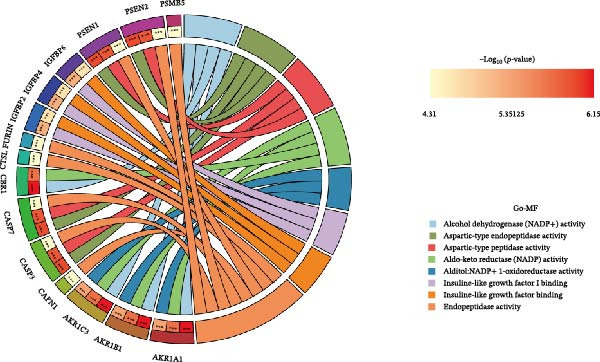
(D)

**Figure:**
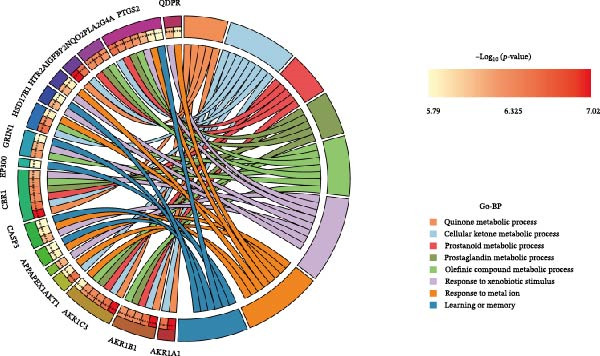
(E)

**Figure:**
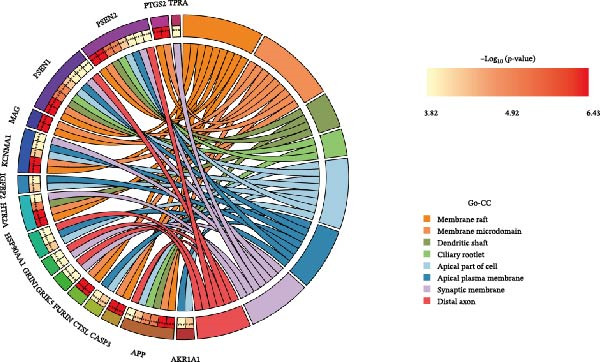
(F)

**Figure:**
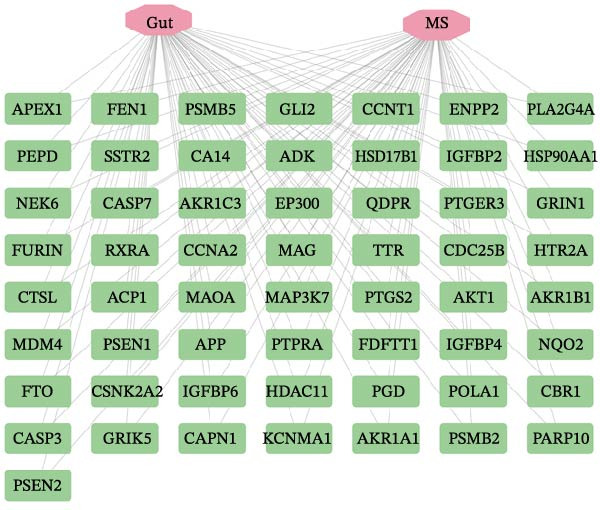
(G)

**Figure:**
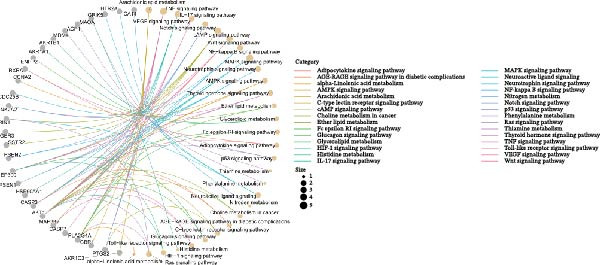
(H)

### 3.2. Functional Enrichment Analysis

In the GO enrichment analysis, the biological process (BP) terms were predominantly enriched in response to xenobiotic stimulus, response to metal ion, and cellular ketone metabolic process (Figure 2D), indicating that the DEGs are closely associated with environmental stress adaptation, metabolic regulation, and neuronal functions. Regarding the cellular component (CC) category, enriched terms included membrane raft, membrane microdomain, and apical part of cell (Figure 2E), suggesting that these DEGs are mainly localized to specialized membrane structures and synaptic regions. For the molecular function (MF) category, significant enrichment was observed in endopeptidase activity, amide binding, and DNA‐binding transcription factor (TF) binding (Figure 2F), highlighting their involvement in enzymatic catalysis, redox reactions, and transcriptional regulation.

In the KEGG pathway enrichment analysis, the significantly enriched signaling pathways mainly included TNF, cAMP, MAPK, IL‐17, Wnt, VEGF, Notch, NF‐κB, neurotrophin, AMPK, thyroid hormone, Ras, calcium, and PI3K‐Akt signaling pathways, as well as arachidonic acid metabolism and neuroactive ligand–receptor interaction (Figure 2H). These pathways are broadly involved in inflammatory responses, apoptosis and cell survival, metabolic regulation, angiogenesis, immune responses, and neural signal transduction, suggesting that gut microbiota metabolism may regulate the development and progression of MS through multiple key signaling networks.

### 3.3. Identification of Key Targets

To identify the core therapeutic targets for the treatment of MS, we submitted 57 overlapping targets to the STRING platform to construct a protein–protein interaction (PPI) network. Subsequently, the CytoHubba plugin in Cytoscape was used to apply five algorithms, namely MCC, MNC, degree, EPC, and radiality, to further identify core targets within the PPI network (Figure 3A–E). As shown in Figure 3F, we selected the top ten targets from each of the five algorithms and obtained their overlapping targets using a Venn diagram. The results revealed that a total of eight genes were identified as core therapeutic targets for MS, namely, AKT1, HSP90AA1, CASP3, EP300, APP, PTGS2, CCNA2, and MAOA.

Figure 3The identification of core targets. (A–E) The identification of core targets using five algorithms from CytoHubba plug‐in in Cytoscape. (F) The core targets acquired from five algorithms using VENN diagram.

**Figure:**
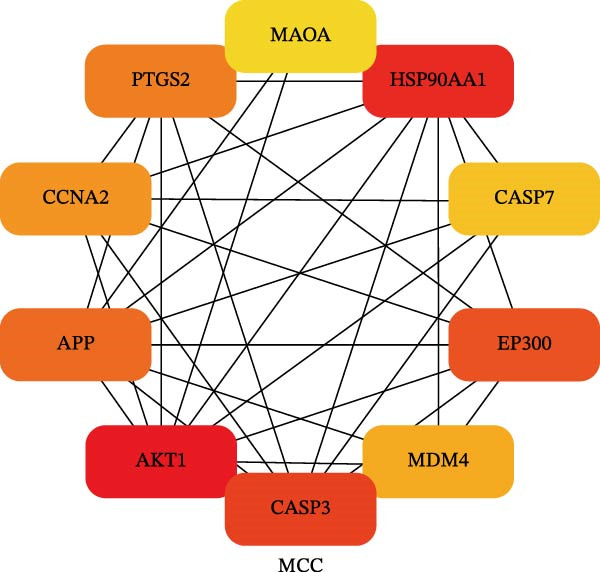
(A)

**Figure:**
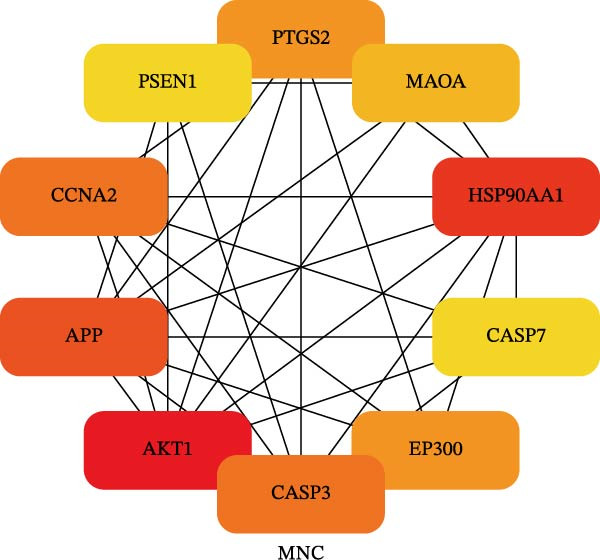
(B)

**Figure:**
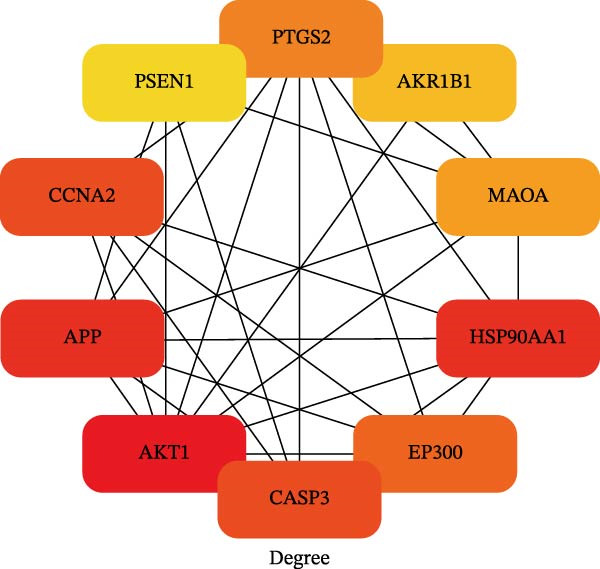
(C)

**Figure:**
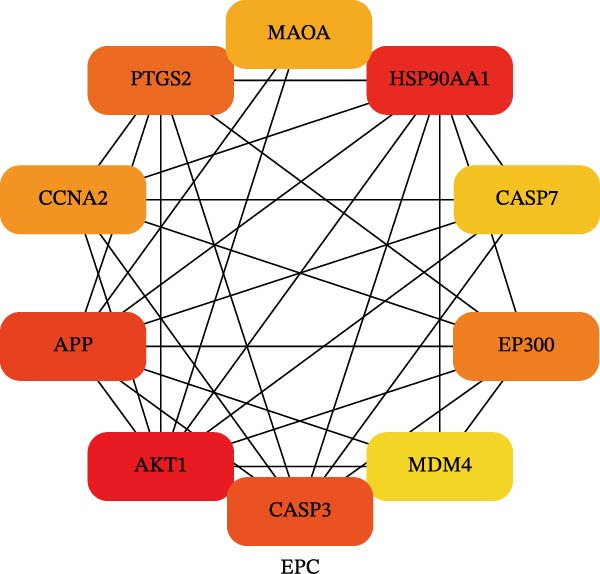
(D)

**Figure:**
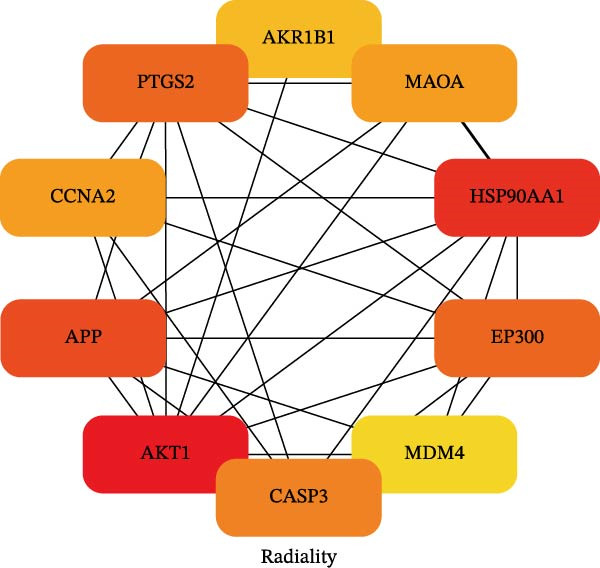
(E)

**Figure:**
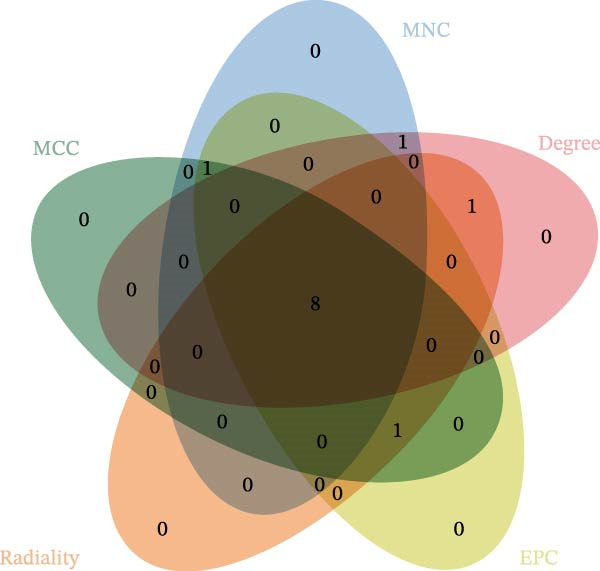
(F)

### 3.4. Identification of Key Predictive Genes via ML

A total of 101 ML models, derived from 110 algorithmic combinations, were assessed using 10‐fold cross‐validation to evaluate their predictive performance. Among these, the elastic net model (*α* = 0.2) exhibited the highest average concordance index (C‐index) and showed robust predictive accuracy in both training and validation datasets (Figure 4A,B). In the training set, the model exhibited perfect fitting characteristics (accuracy = 1, precision = 1, recall = 1, F1 = 1), indicating its ability to accurately distinguish MS from control samples. However, in external validation datasets (such as GSE108000 and GSE21942), the model’s classification performance declined. Among these, GSE108000 showed relatively good performance (accuracy = 0.9, precision = 1, recall = 0.882, F1 = 0.938), whereas GSE21942 performed moderately (accuracy = 0.517, precision = 1, recall = 0.5, F1 = 0.667), suggesting that the model’s generalizability is limited in certain external datasets. Overall, these metrics provide a multidimensional evaluation of the model’s predictive performance, offering a reliable reference for subsequent analyses.

Figure 4Core target screening by ML. (A) Heatmap of ML results; (B) ROC curve evaluating model accuracy; (C) intersection of core targets screened by the optimal machine learning algorithm and targets identified through PPI analysis; (D) boxplot of DEGs; (E) ROC curve evaluating the diagnostic performance of candidate genes; (F) relationships among the three genes; and (G) chromosomal locations of the intersecting genes labeled on the corresponding chromosomes.  ^∗^ *p* < 0.05,  ^∗∗^ *p* < 0.01,  ^∗∗∗^ *p* < 0.001.

**Figure:**
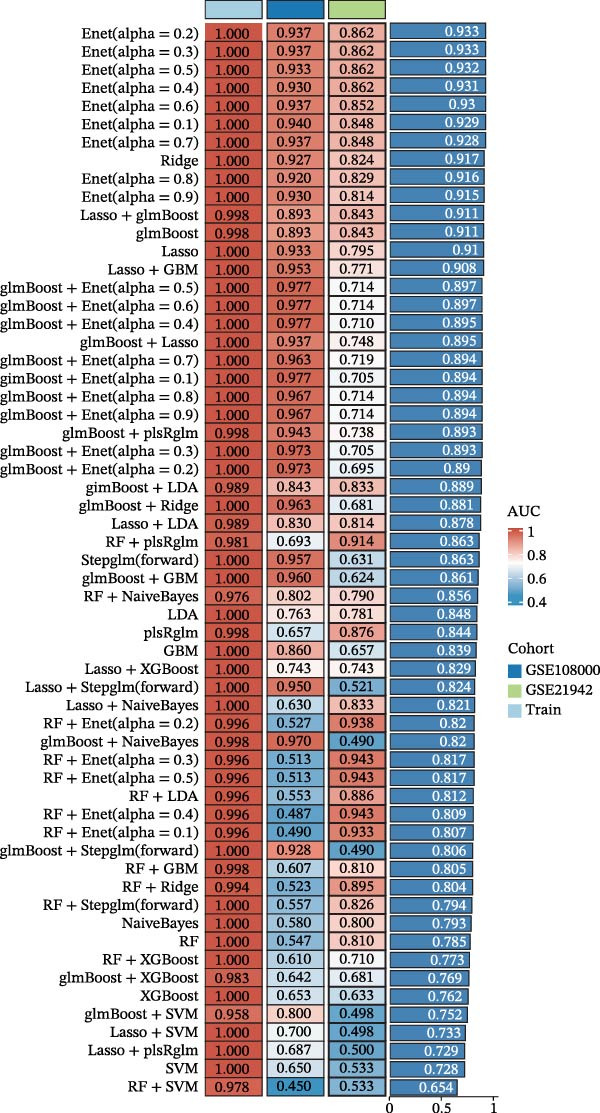
(A)

**Figure:**
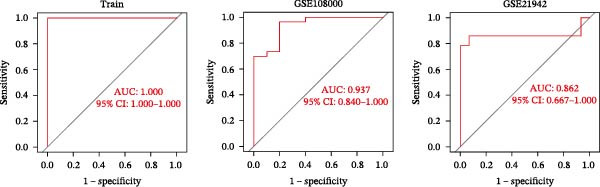
(B)

**Figure:**
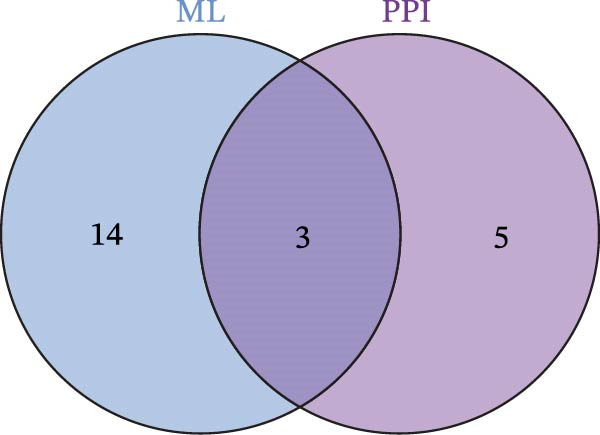
(C)

**Figure:**
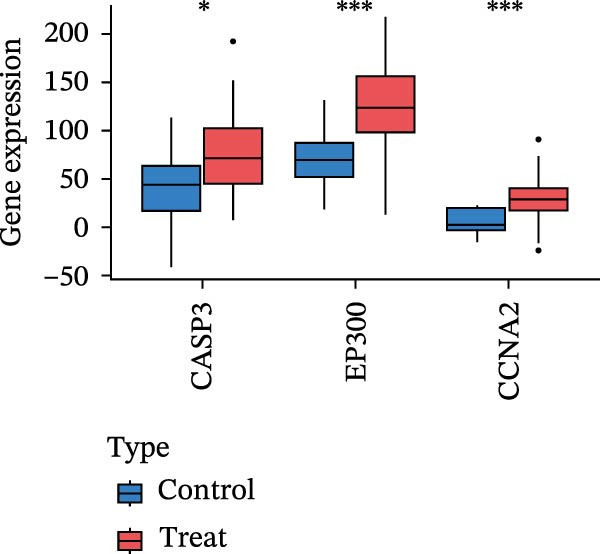
(D)

**Figure:**
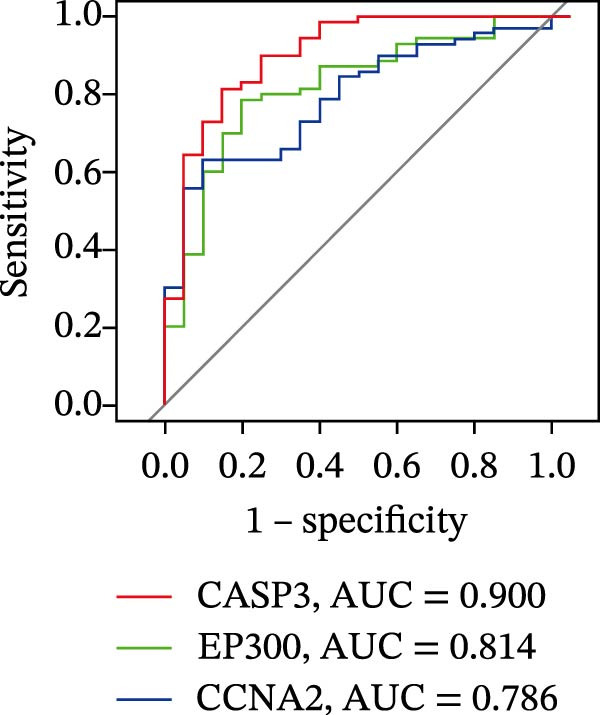
(E)

**Figure:**
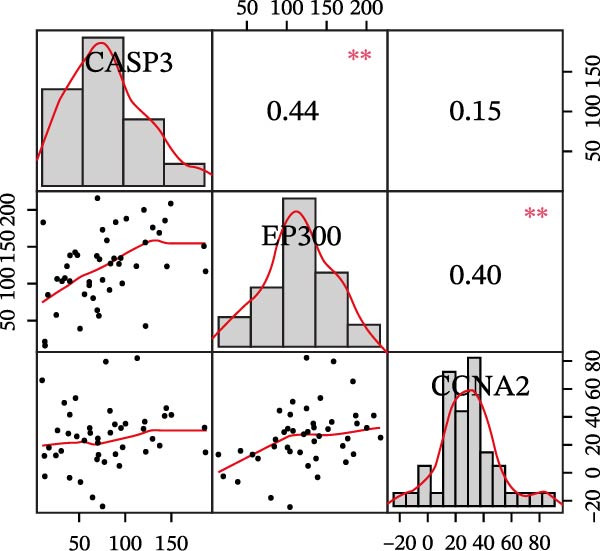
(F)

**Figure:**
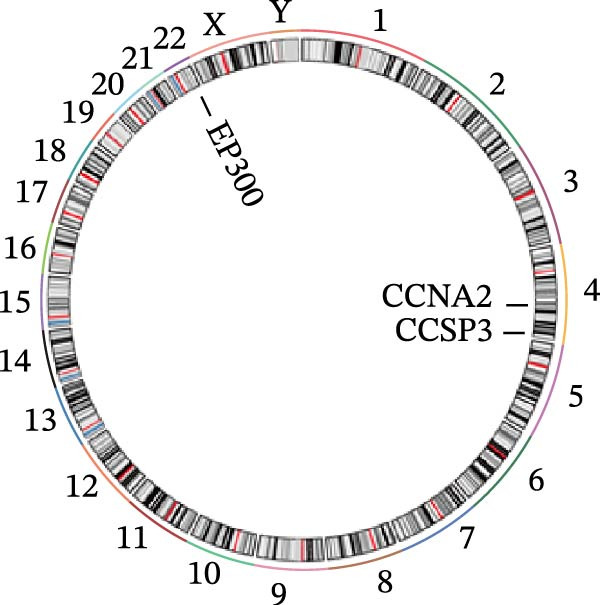
(G)

To pinpoint key regulatory genes, results from the ML analysis were intersected with CytoHubba predictions, yielding three core targets: EP300, CCNA2, and CASP3 (Figure 4C). We observed upregulation of the three core genes through boxplot analysis (Figure 4D) and plotted their ROC curves to assess discriminatory accuracy (Figure 4E). In addition, we analyzed the correlations among these three genes (Figure 4F) and employed a circos plot to visualize their interrelationships and chromosomal distributions (Figure 4G).

### 3.5. SHAP Analysis for Model Interpretation

To evaluate overall diagnostic performance, a ROC curve was plotted (Figure 5A). SHAP analysis indicated that CASP3, EP300, and CCNA2 play key roles in model predictions, with CASP3 contributing the most and EP300 the least (Figure 5B). Partial dependence analysis showed a monotonic positive correlation between gene expression and predictive contribution, consistent with a “dose‐dependent” risk pattern (Figure 5C). The positive SHAP values of these three genes further confirmed that their high expression is a risk factor for adverse outcomes (Figure 5D). Moreover, CASP3 exhibited potential synergistic effects with EP300 and CCNA2: when EP300 or CCNA2 expression was high, the SHAP value of CASP3 increased, suggesting joint effects in promoting poor outcomes. Overall, SHAP analysis made the model interpretation more intuitive and quantified each gene’s impact, while gene expression patterns could distinguish patients across samples, supporting comprehensive model interpretation.

Figure 5SHAP analysis and pQTL‐based MR analysis. (A) ROC curves constructed using ten machine learning models to evaluate overall diagnostic performance. (B) Bar chart with the vertical axis representing gene names and the horizontal axis representing the mean absolute SHAP value. (C) To display the prediction results for a single sample, a baseline value was first established, followed by predictions for each gene. (D) Bee swarm plot with the vertical axis representing gene names and the horizontal axis representing SHAP values, used to calculate the mean SHAP value for each gene. (E) Scatter plot with gene expression on the *x*‐axis and the corresponding SHAP value on the *y*‐axis. (F) MR analysis of the CASP3 gene, including scatter plot, funnel plot, and leave‐one‐out analysis plot.

**Figure:**
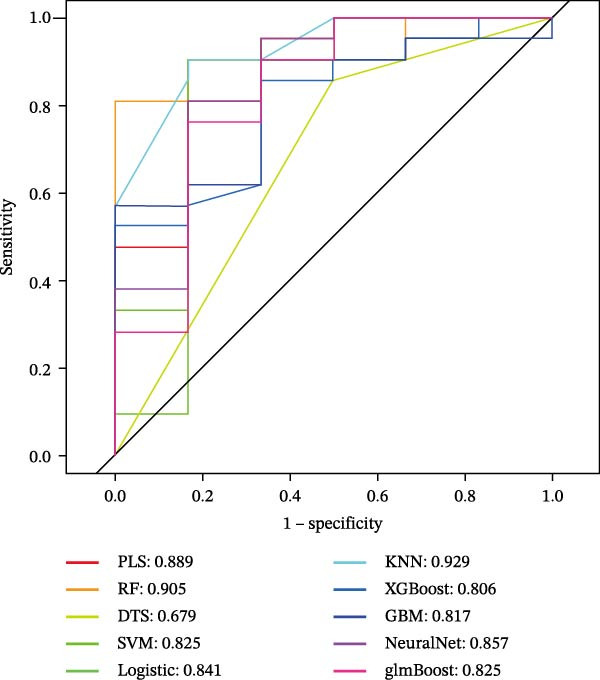
(A)

**Figure:**
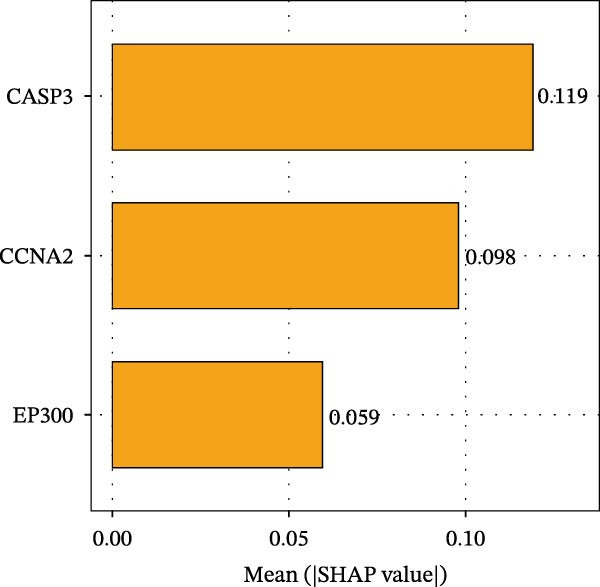
(B)

**Figure:**
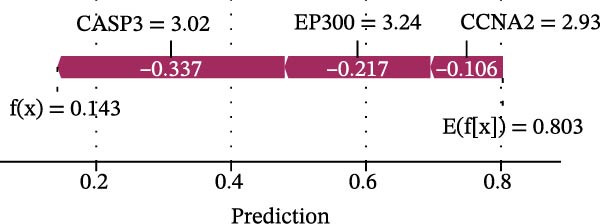
(C)

**Figure:**
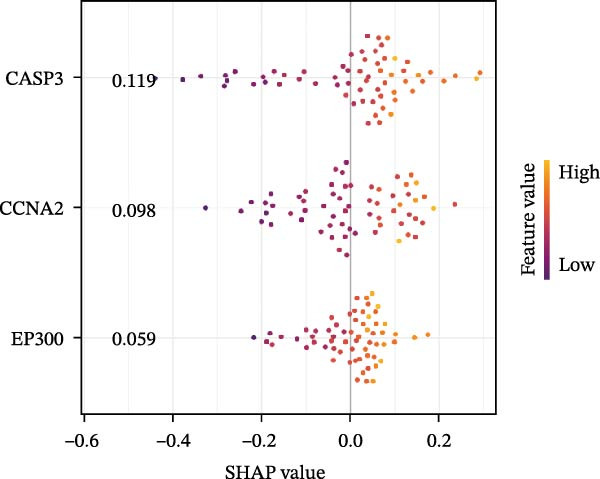
(D)

**Figure:**
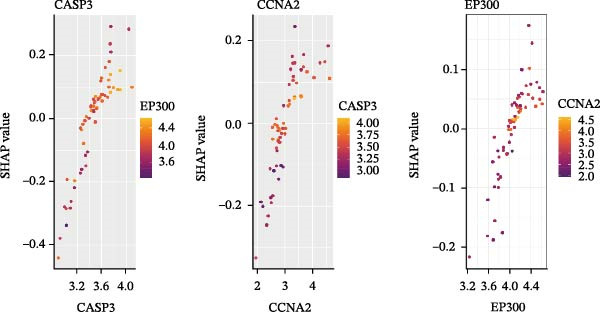
(E)

**Figure:**
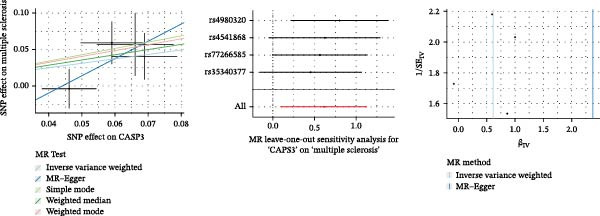
(F)

### 3.6. MR Identifies Genetically Causal Targets

MR analysis based on protein quantitative trait loci (pQTL) was performed to assess genetic causality. Using the IVW method as the primary criterion, among the three core genes, only CASP3 showed a significant genetic association with MS (*β* = 0.611, SE = 0.265, *p* = 0.021), with an odds ratio (OR) of 1.84 (95% CI: 1.10–3.10), supporting its potential as a therapeutic target (Figure 5F). Sensitivity analyses indicated robust results: the MR–Egger intercept did not suggest horizontal pleiotropy (*β* = −0.105, SE = 0.099, *p* = 0.402), Cochran’s *Q* test showed no significant heterogeneity (IVW *Q* = 2.21, *p* = 0.53), and the MR–PRESSO global test detected no significant pleiotropy (RSSobs = 3.75, *p* = 0.574). These results suggest that the association between CASP3 and MS based on IVW is unlikely to be affected by pleiotropy or heterogeneity, further reinforcing CASP3 as a key causal gene.

### 3.7. CASP3 Brain Expression, Subcellular Localization, and Functional Pathway Analysis

We analyzed the expression of CASP3 in the brain and found that it was highly expressed in the cerebellum (Figure 6A). Further spatial transcriptomics analysis indicated that CASP3 was highly expressed in astrocytes, whereas no expression was detected in microglia (Figure 6B). Subcellular localization analysis revealed that CASP3 was primarily localized in the nucleoplasm and mitochondria (Figure 6C).

Figure 6Expression patterns and functional enrichment analysis of CASP3. (A) CASP3 brain RNA expression. (B) CASP3 spatial analysis by STEREO‐seq. (C) CASP3 subcellular localization analysis. (D) GeneMainA analysis. (E) GSEA functional enrichment. (F) GSVA functional enrichment.

**Figure:**
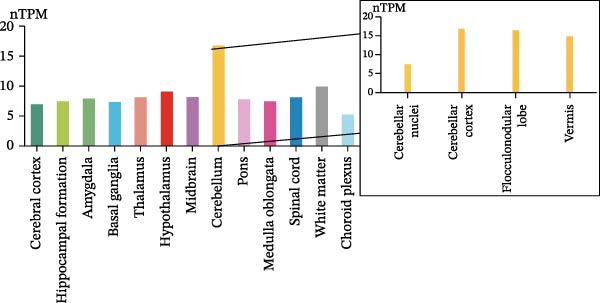
(A)

**Figure:**
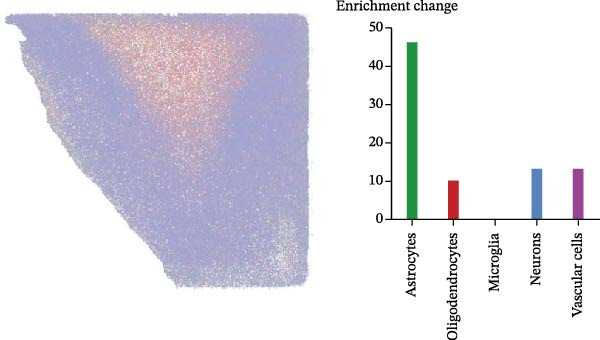
(B)

**Figure:**
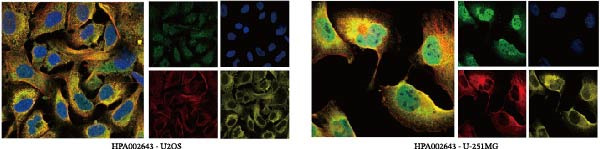
(C)

**Figure:**
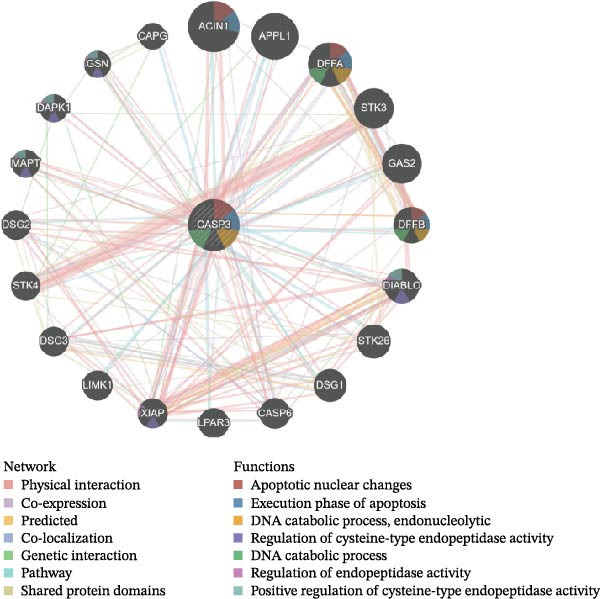
(D)

**Figure:**
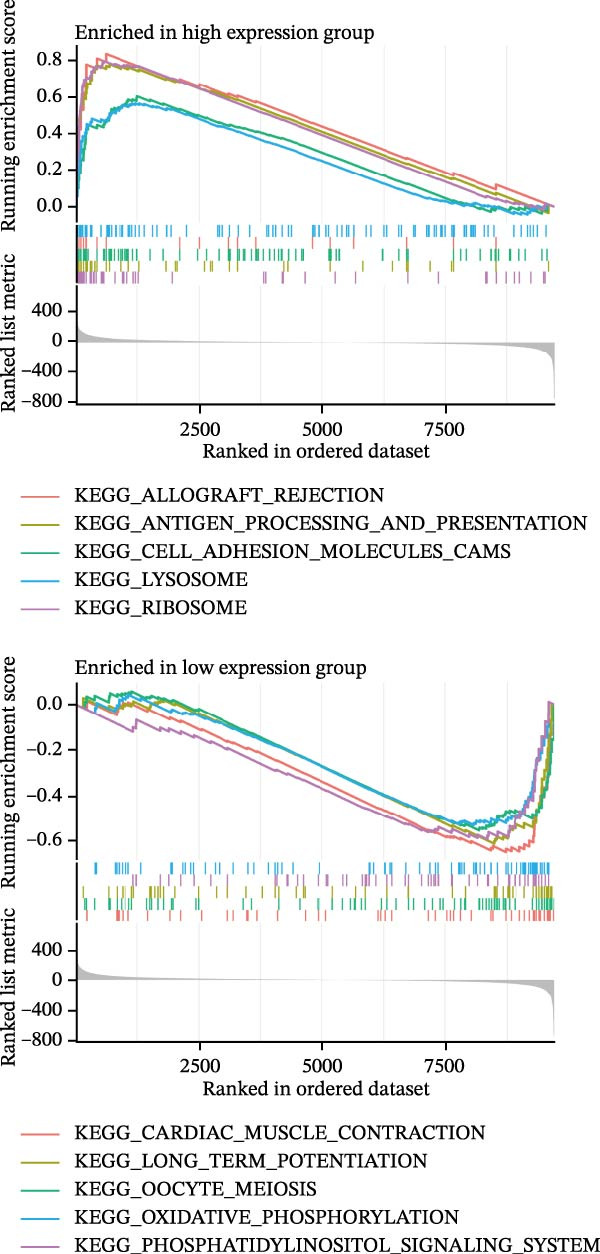
(E)

**Figure:**
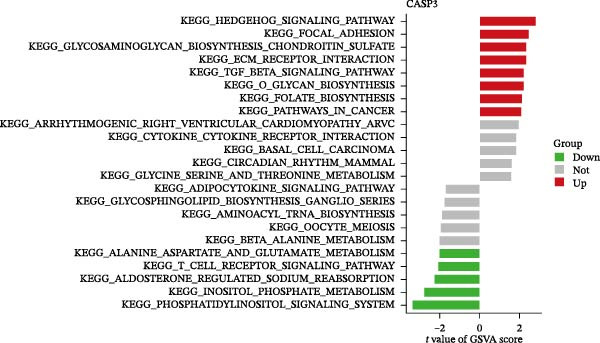
(F)

In addition, we used GeneMANIA, GSEA, and GSVA to analyze the functions and enriched pathways of the high‐ and low‐expression groups of the target genes, and visualized the most significantly enriched pathways (Figure 6E,F).

### 3.8. Construction of miRNA–TF–mRNA Regulatory Network

To explore the upstream regulatory mechanisms of CASP3, we performed predictive analyses to identify relevant miRNAs and TFs. First, TFs were predicted using the TRRUST and hTFtarget databases, and intersection analysis yielded four TFs (Figure 7A–C). Meanwhile, forty nine miRNAs were predicted using mirWalk and miRDB (Figure 7D). We then constructed an integrated regulatory network comprising forty nine miRNAs, four TFs, and CASP3 (Figure 7E). This network highlights key regulators that may influence CASP3 expression and provides new insights into its molecular interactions in the pathogenesis of MS.

Figure 7Construction of the miRNA–TF–mRNA network. (A) TFs predicted using the TRRUST database. (B) TFs predicted using the hTFtarget database. (C) Venn diagram showing three TFs of CASP3. (D) The miRNAs targeting CASP3 predicted using miRDB and miRWalk databases. (E) The miRNA–TF–mRNA network, in which yellow nodes represent miRNAs, red nodes represent mRNAs, and purple nodes represent TFs.

**Figure:**
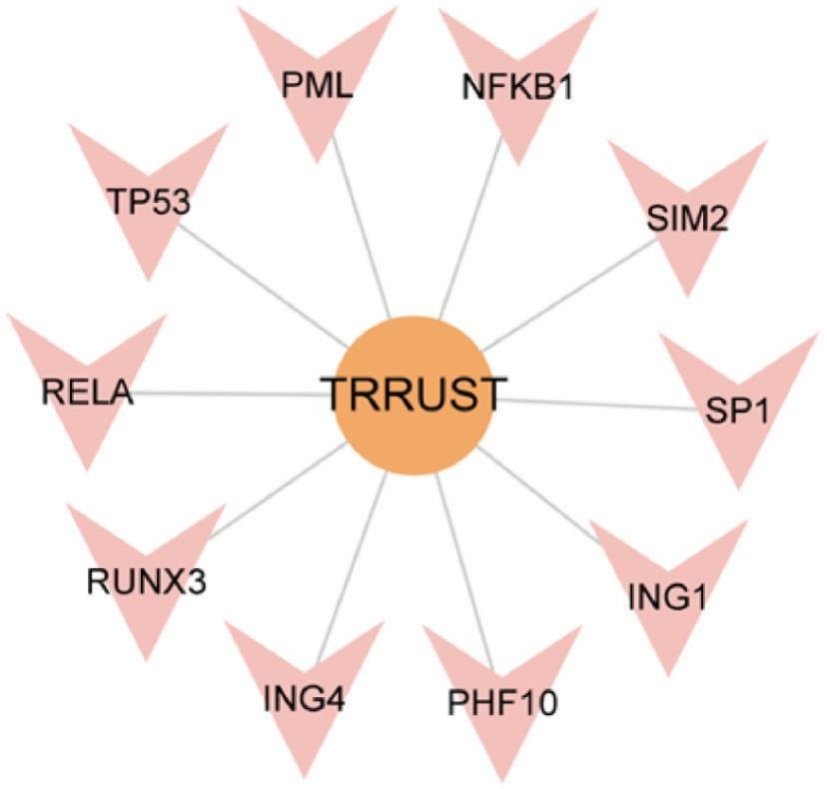
(A)

**Figure:**
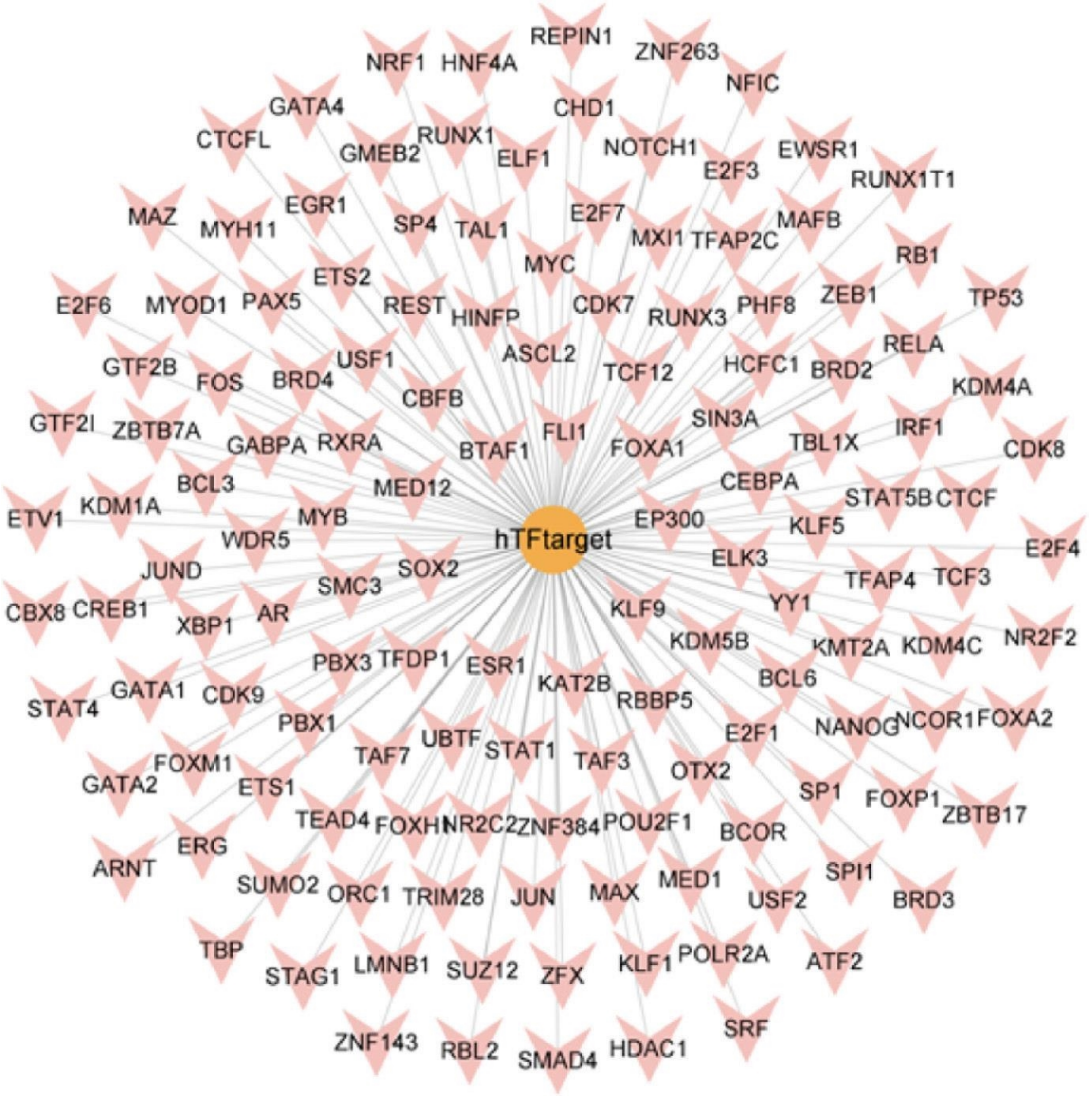
(B)

**Figure:**
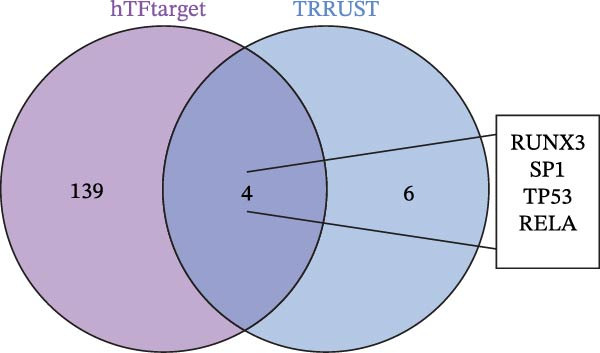
(C)

**Figure:**
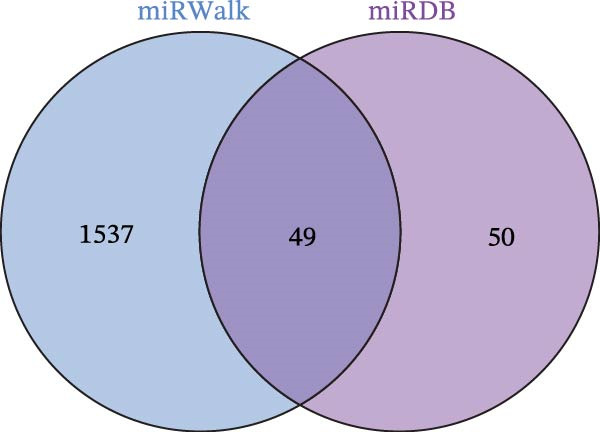
(D)

**Figure:**
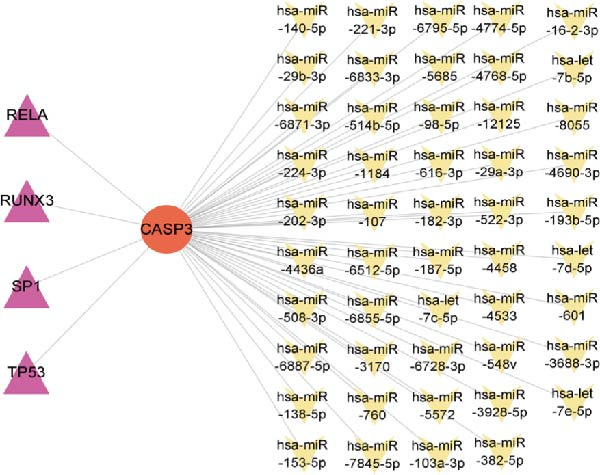
(E)

### 3.9. Cell–Cell Communication Analysis in CSF Single Cells

Single‐cell RNA‐seq data from the CSF of six MS patients were subjected to quality control, dimensionality reduction, and clustering analysis. Cells were annotated into four major immune cell types, namely T cells, monocytes, NK cells, and B cells, using SingleR (Figure 8A–F). CASP3 was expressed across all cell subtypes, with predominant expression in T cells and B cells (Figure 8G,H).

Figure 8Based on single‐cell RNA‐seq data, different annotated cell clusters in MS were identified. (A) Quality control of scRNA‐seq data. (B) Variance plot showing gene expression variability across all cells. (C) PCA revealing clear separation of cells. (D) PCA identified the top 20 principal components (PCs) with *p* < 0.05, and the tSNE algorithm was applied to these PCs for dimensionality reduction. (E) tSNE successfully classified 16 cell subpopulations. (F) All 4 cell clusters were annotated using SingleR. (G) Expression distribution of CASP3. (H) Violin plot showing CASP3 expression across different cell subtypes.

**Figure:**
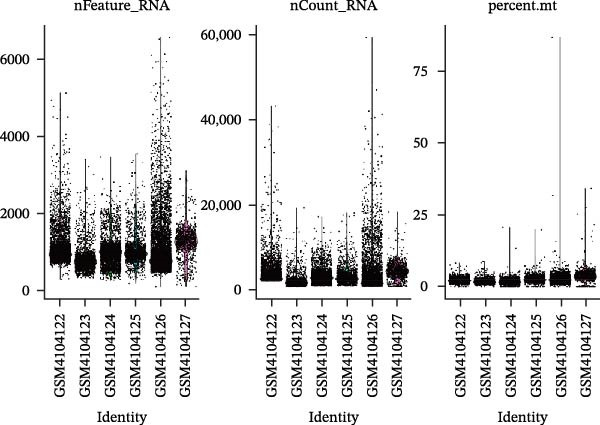
(A)

**Figure:**
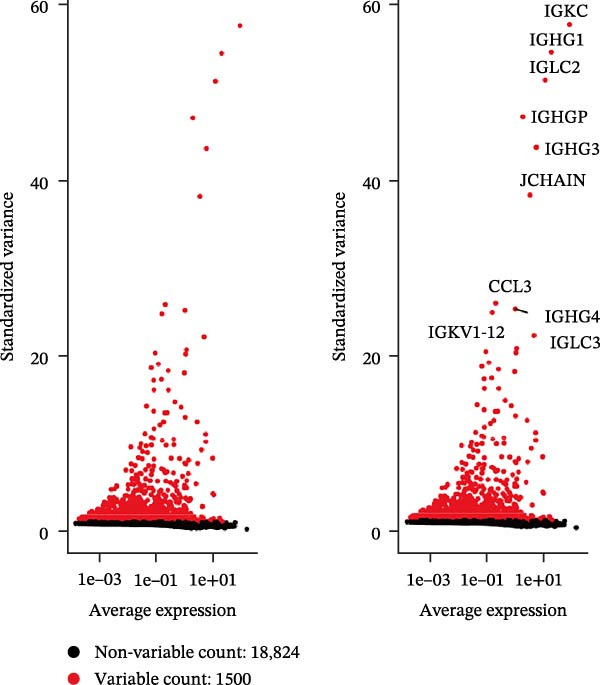
(B)

**Figure:**
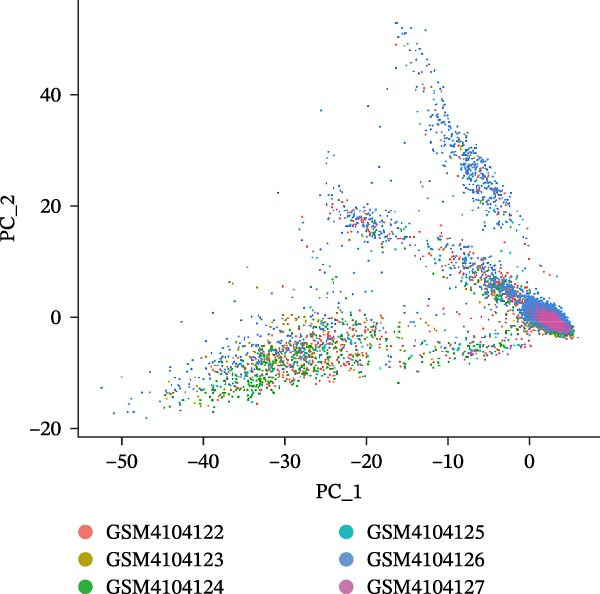
(C)

**Figure:**
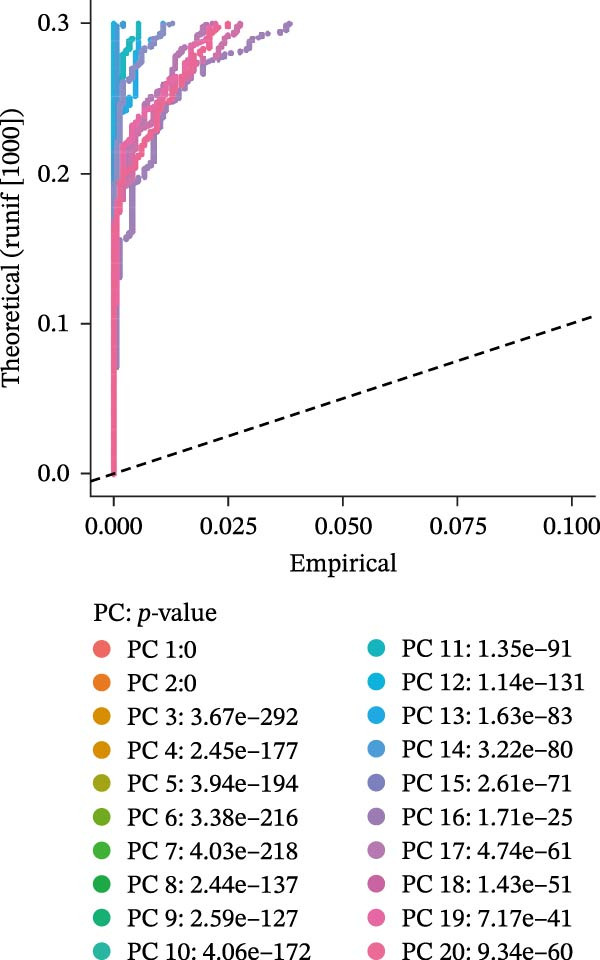
(D)

**Figure:**
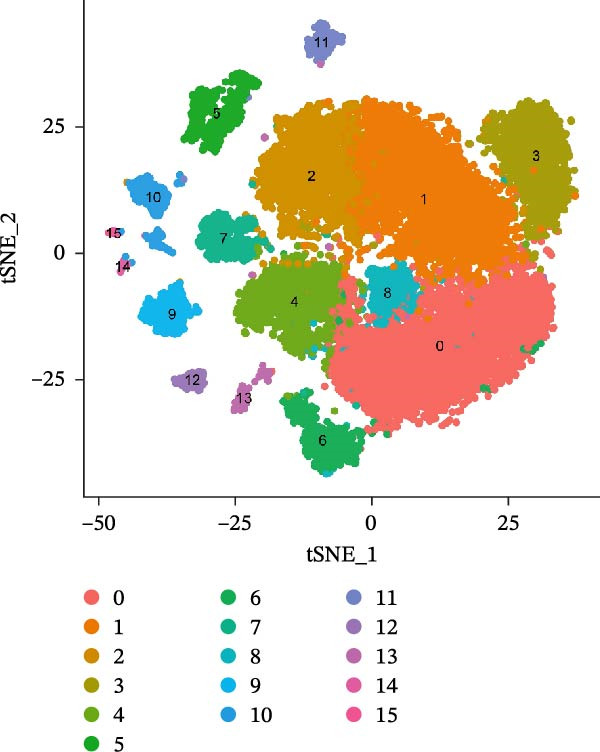
(E)

**Figure:**
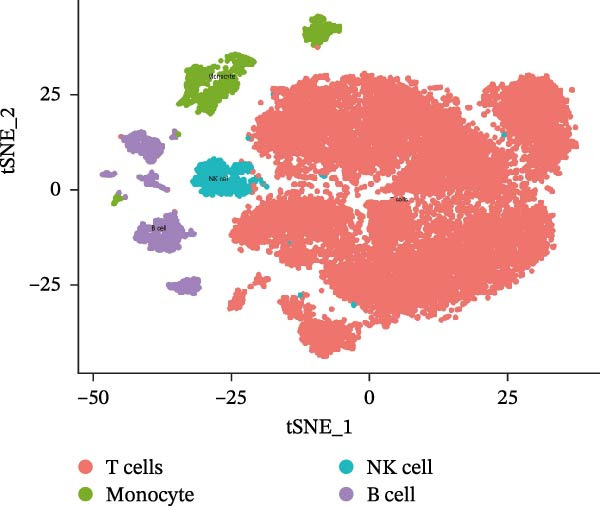
(F)

**Figure:**
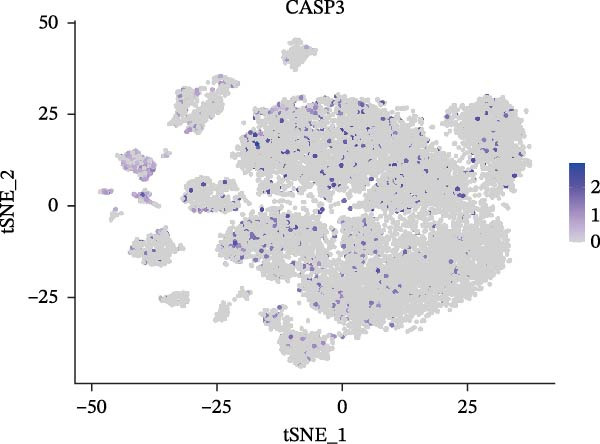
(G)

**Figure:**
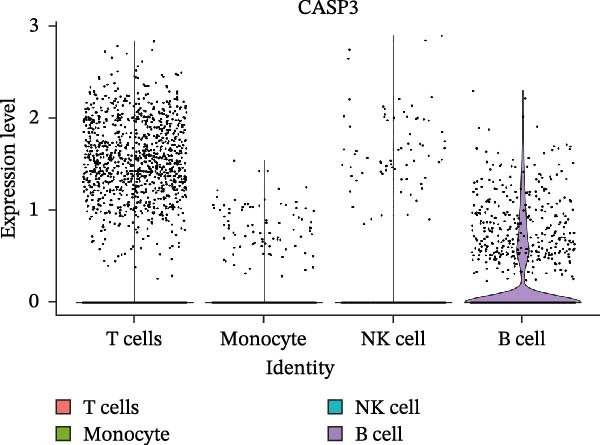
(H)

Following identification of DEGs, intercellular communication networks were reconstructed. The galectin signaling pathway emerged as a central regulatory axis, with monocytes acting as the primary signal source and NK and T cells as the main targets (Figure 9A–D). Signal propagation occurred predominantly through the LGALS9–CD44 and LGALS9–CD45 ligand–receptor interactions (Figure 9E). Importantly, monocytes influence the other three cell populations via these ligand‐receptor pairs, while NK cells, T cells, and the remaining cell type do not appear to communicate with one another through the galectin pathway (Figure 9F,G).

Figure 9Cell–cell communication analysis of MS cell subsets with distinct differentiation patterns. (A) Dot plot of ligand–receptor (L–R) pairs in subtype‐specific pathways. (B) Cell–cell communication bubble plot. (C) Network centrality scoring of the galectin signaling pathway. (D) Heatmap of the galectin signaling pathway. (E) Contribution of individual receptors to the overall galectin signaling pathway. (F) Expression distribution of galectin pathway‐related genes. (G) Co‐expression of receptors across different cell types in the galectin signaling pathway.

**Figure:**
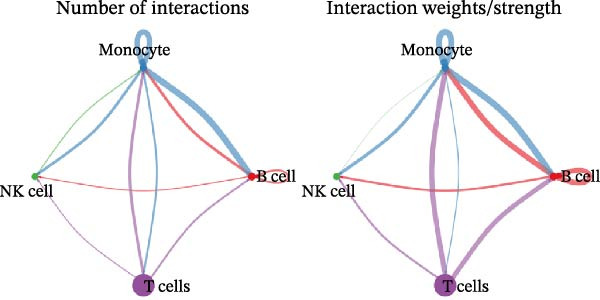
(A)

**Figure:**
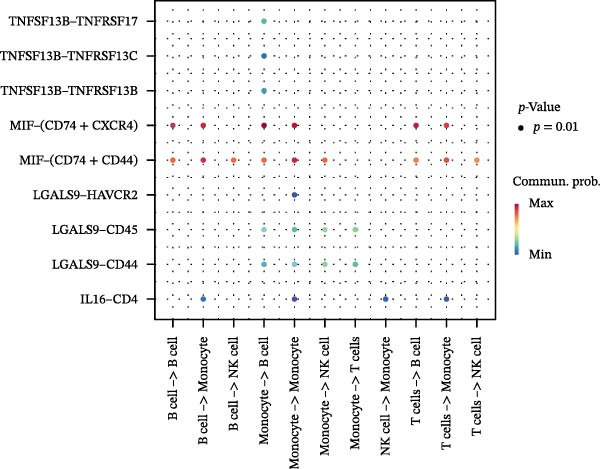
(B)

**Figure:**
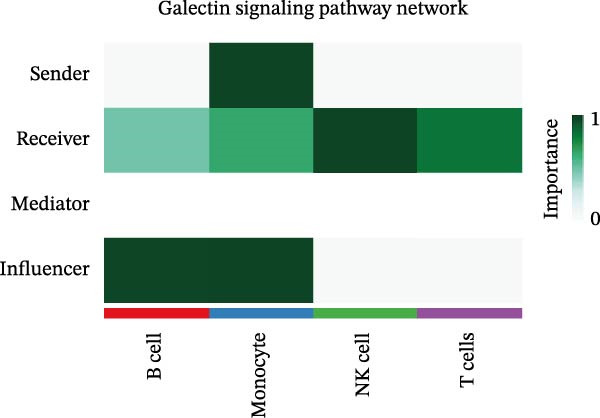
(C)

**Figure:**
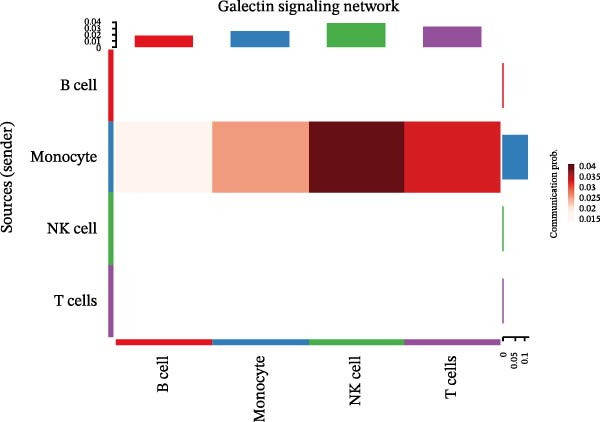
(D)

**Figure:**
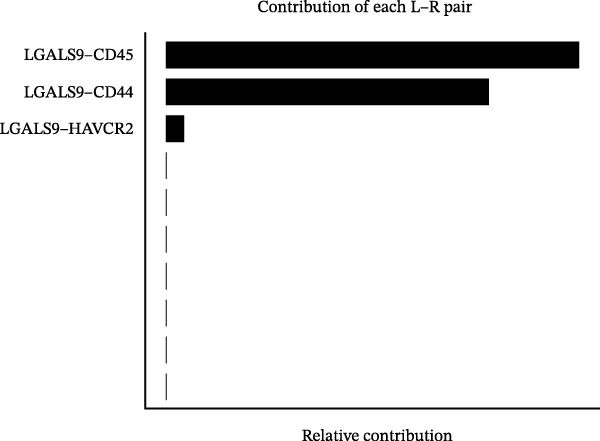
(E)

**Figure:**
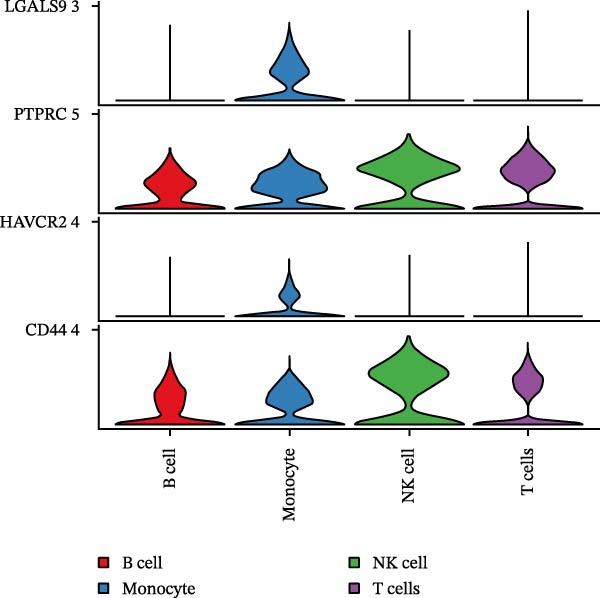
(F)

**Figure:**
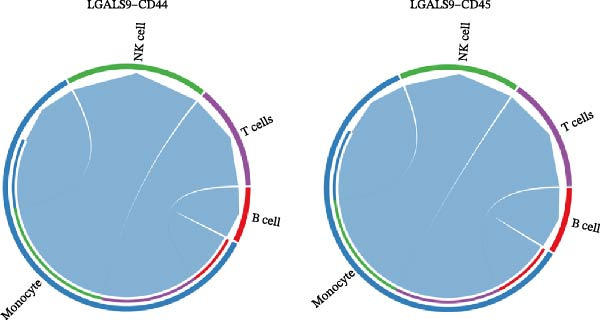
(G)

### 3.10. Identification of Core Gut Microbiota Metabolites

**Figure 10:**
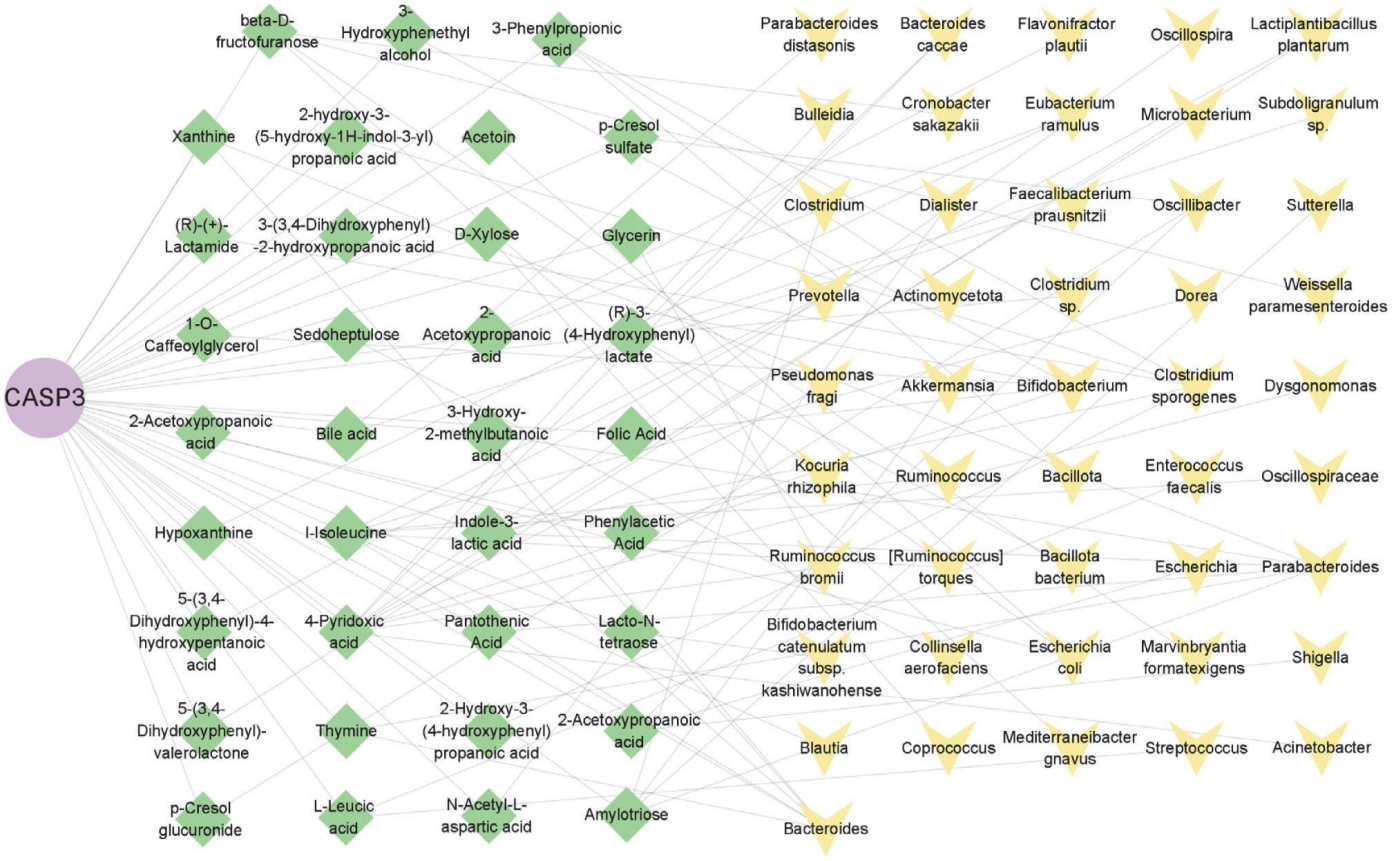
Gut microbiota–metabolite–target network. Purple nodes represent target genes, green nodes represent microbial metabolites, and yellow nodes represent gut microbiota.

To identify the core gut microbiota‐derived metabolites involved in the treatment of MS, we constructed a gut–microbiota–metabolite–target (G–M–T) network to illustrate the complex relationships among them. As shown in Figure 10, the network comprised one target, thirty five metabolites, and forty six gut microbial species. Notably, l‐isoleucine and 3‐hydroxy‐2‐methylbutanoic acid showed extensive associations with gut microbiota, while microbial species, such as *Bacteroides*, *Bifidobacterium*, *Parabacteroides*, *Clostridium sporogenes*, and *Escherichia coli*, were closely linked to these metabolites. These findings suggest that these metabolites and gut microbes may play functional roles in the regulation of MS.

### 3.11. Assessment of Drug‐Likeness and Safety of Metabolites

To screen metabolites with potential therapeutic value, we conducted a systematic analysis of their drug‐likeness and safety profiles. Molecular properties were evaluated using Lipinski’s Rule of Five, and potential toxicities were predicted based on toxicological parameters. The results indicated that nine metabolites not only met the drug‐likeness criteria but also showed no predicted hepatotoxicity, nephrotoxicity, carcinogenicity, or neurotoxicity (Tables 1 and 2), suggesting their potential as safe candidates for drug development.

**Table 1:** Evaluation of drug‐like properties of core metabolites.

Compound	MW (g/mol)	HBA	HBD	M Log *P*	Lipinski’s violations	Bioavailability score	TPSA
3‐Hydroxy‐2‐methylbutanoic acid	118.13	3	2	0.01	0	0.85	57.53
L‐isoleucine	131.17	3	2	−1.82	0	0.55	63.32
(R)‐(+)‐Lactamide	89.09	2	2	−1.25	0	0.55	63.32
1‐O‐caffeoylglycerol	254.24	6	4	−0.07	0	0.55	107.22
3‐Hydroxyphenethyl alcohol	138.16	2	2	1.21	0	0.55	40.46
2‐Hydroxy‐3‐(4‐hydroxyphenyl) propanoic acid	182.17	4	3	0.52	0	0.56	77.76
3‐(3,4‐Dihydroxyphenyl)‐2‐hydroxypropanoic acid	198.17	5	4	−0.04	0	0.56	97.99
D‐xylose	150.13	5	4	−2.32	0	0.55	90.15
5‐(3,4‐Dihydroxyphenyl)‐4‐hydroxypentanoic acid	226.23	5	4	0.55	0	0.56	97.99

**Table 2:** Assessment of toxicity for core metabolites.

Compound	hERG blockers	H‐HT	DILI	Nephrotoxicity	Neurotoxicity	Carcinogenicity	LD50 (mg/L)
3‐Hydroxy‐2‐methylbutanoic acid	Non‐blocker	–	–	–	–	–	3.322
L‐isoleucine	Non‐blocker	–	–	–	–	–	3.874
(R)‐(+)‐Lactamide	Non‐blocker	–	–	–	–	–	2.607
1‐O‐caffeoylglycerol	Non‐blocker	–	–	–	–	–	4.859
3‐Hydroxyphenethyl alcohol	Non‐blocker	–	–	–	–	–	3.528
2‐Hydroxy‐3‐(4‐hydroxyphenyl) propanoic acid	Non‐blocker	–	–	–	–	–	3.474
3‐(3,4‐Dihydroxyphenyl)‐2‐hydroxypropanoic acid	Non‐blocker	–	–	–	–	–	3.714
D‐xylose	Non‐blocker	–	–	–	–	–	2.058
5‐(3,4‐Dihydroxyphenyl)‐4‐hydroxypentanoic acid	Non‐blocker	–	–	–	–	–	3.582

*Note:* LD_50_, 48‐h Daphnia magna 50% lethal concentration (LC_50_); LC_50_, values are expressed as −log_10_[(mg/L)/(1000 × molecular weight)]; “–”, indicates a negative result.

Abbreviations: DILI, drug induced liver injury; H‐HT, human hepatotoxicity; LC, lethal concentration.

### 3.12. Molecular Docking

Molecular docking results showed that among these nine metabolites, eight exhibited binding energies below −5 kcal/mol with CASP3, indicating that CASP3 can stably bind to these eight nontoxic metabolites with drug development potential. Notably, (R)‐(+)‐lactamide exhibited the lowest binding energy at −3.9 kcal/mol, while 3‐(3,4‐dihydroxyphenyl)‐2‐hydroxypropanoic acid had the highest at −6.3 kcal/mol (Figure 11). These findings suggest that these metabolites may exert biological effects through interactions with CASP3 and hold potential therapeutic value.

Figure 11Molecular docking results. (A–I) Docking of CASP3 with nine nontoxic, druggable metabolites.

**Figure:**
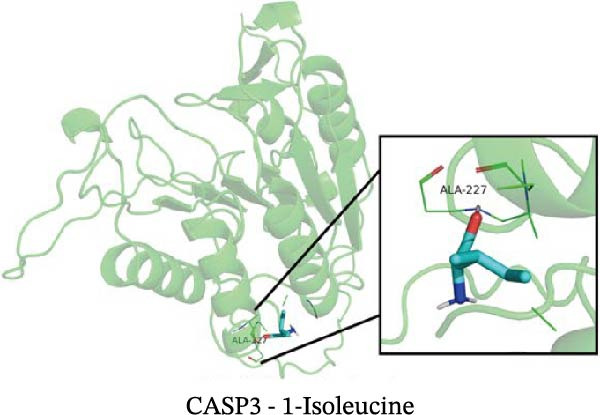
(A)

**Figure:**
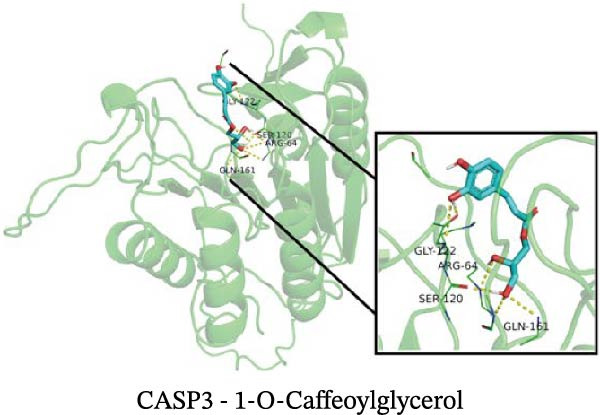
(B)

**Figure:**
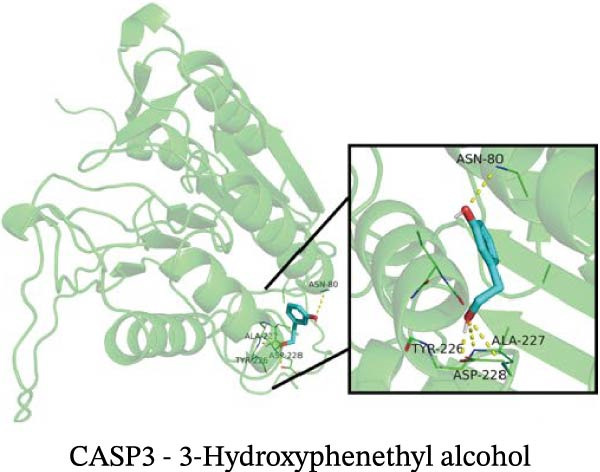
(C)

**Figure:**
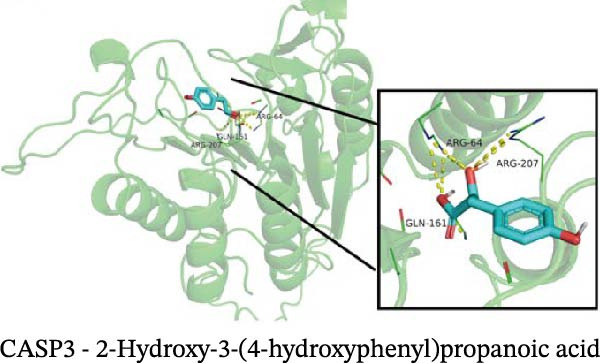
(D)

**Figure:**
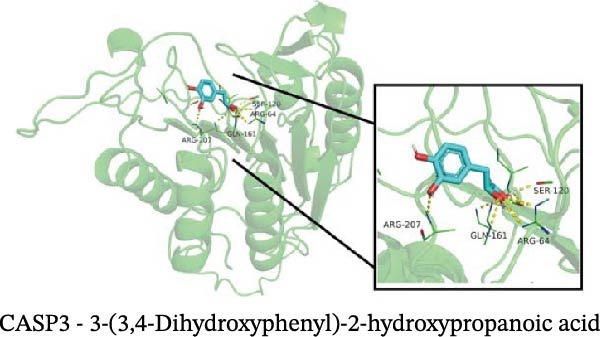
(E)

**Figure:**
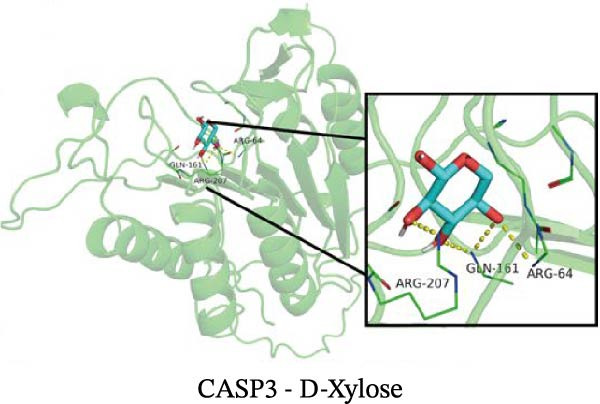
(F)

**Figure:**
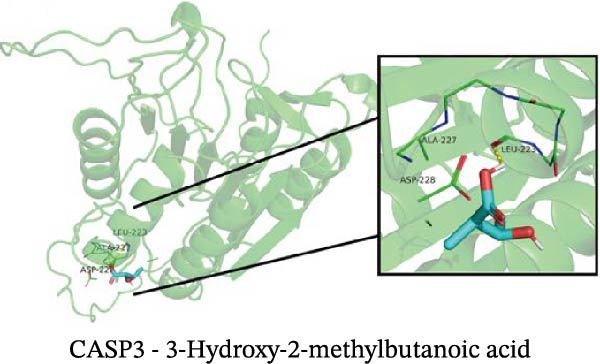
(G)

**Figure:**
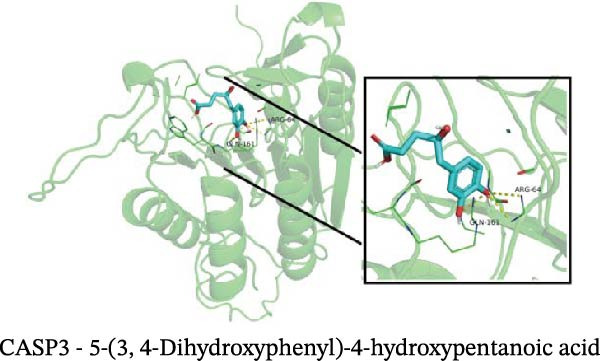
(H)

**Figure:**
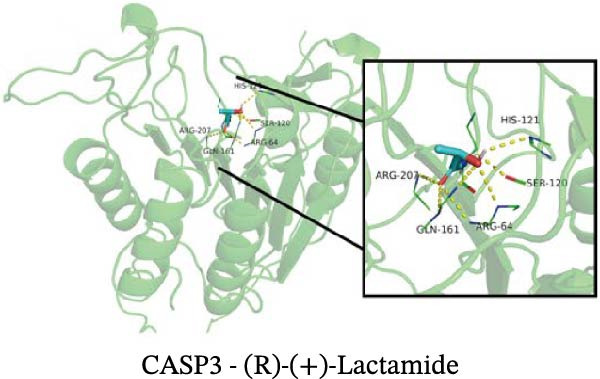
(I)

## 4. Discussion

MS is a chronic autoimmune disease affecting the central nervous system, characterized by recurrent episodes of neurological dysfunction and progressively worsening disability [22]. In recent years, an increasing number of studies have shown that the gut microbiota plays a crucial role in the onset and progression of MS [23], providing new insights for early diagnosis and intervention. Therefore, this study integrated network pharmacology, ML, SHAP analysis, and MR analysis to identify CASP3 as a core target, and to systematically explore the potential immunoregulatory associations between gut microbial metabolites and key targets in MS through G–M–T networks and single‐cell transcriptomic analysis.

CASP3 is considered to be closely associated with programmed cell death and inflammation in MS and its animal models. Studies have shown that CASP3 activation can be detected in oligodendrocytes within EAE lesions, suggesting its involvement in oligodendrocyte apoptosis and the promotion of demyelinating pathology [24]. In addition, CSF from MS patients has been found to induce neuronal apoptosis and activate CASP3, while inhibition of caspase activity in vitro can attenuate such damage, indicating a potential therapeutic target in neuronal injury [25]. Recent findings further reveal that activation of CASP3/7 in microglia can trigger pyroptosis‐like inflammatory cell death, thereby amplifying central nervous system inflammation and conferring a proinflammatory role to CASP3 [26]. Moreover, inhibition of the inflammasome/caspase pathway (e.g., CASP1) has been shown to reduce inflammation and demyelination in EAE, suggesting that targeting the caspase network as a whole may provide protective effects, though the outcomes likely depend on cell type and disease stage, requiring careful evaluation [27]. Collectively, current evidence supports the notion that CASP3 contributes both to neuronal and oligodendrocyte apoptosis and to the amplification of inflammation in MS. However, its role in the regulation of MS pathogenesis via the gut–CNS axis remains unclear and warrants further investigation.

Enrichment analysis indicated that gut microbiota metabolites may act through several MS‐related signaling pathways. The TNF pathway can promote inflammation and participate in repair [28]. The cAMP pathway regulates immune and neural cell functions and may have anti‐inflammatory and neuroprotective effects [29]. The MAPK pathway is involved in immune cell activation and demyelination [30]. The IL‐17 pathway mediates inflammation in astrocytes and oligodendrocytes via Th17/IL‐17 [31]. The Wnt pathway regulates oligodendrocyte differentiation and remyelination, and its disruption may impair repair [32]. The VEGF pathway affects the blood–brain barrier and neuroprotection, with bidirectional roles in MS [33]. These findings suggest that gut microbiota metabolites may coordinate MS‐related inflammation and neural repair through key signaling networks.

In this study, we screened 251 potential gut microbiota‐derived metabolites from the gutMGene database and performed intersection analysis between their predicted target genes and MS‐related DEGs, thereby identifying key metabolites highly associated with core targets in the G–M–T network, such as CASP3. Based on network connectivity and drug‐likeness criteria, nine small‐molecule metabolites were prioritized for further investigation due to their favorable drug‐like properties and low predicted toxicity. In the G–M–T network, the metabolites L‑isoleucine and 3‑hydroxy‑2‑methylbutanoic acid are typical products of branched‐chain amino acid (BCAA) synthesis and catabolism, catalyzed by the acetohydroxyacid synthase complex (AHAS), which has been extensively studied in *Escherichia coli*. The ilv operons in *E. coli* are feedback‐regulated by BCAAs, and different AHAS isoenzymes show varying sensitivity to isoleucine, valine, and leucine, providing a potential mechanism for the observed variation in these metabolites [34]. The gut bacterium *Akkermansia muciniphila*, on the other hand, degrades mucin O‑glycans via glycosidases and sulfatases [35], producing SCFAs during fermentation and promoting SCFA production by other gut bacteria [36]. These mechanisms may offer a potential explanation for the microbe‐metabolite associations observed in the G–M–T network.

Further analysis indicated that these metabolites may be functionally relevant to the development and progression of MS. For example, the BCAA L‐isoleucine is elevated in the plasma of MS patients, potentially reflecting abnormal energy metabolism and correlating with neurofilament light chain levels, suggesting a possible link to neuronal damage or disease activity [37, 38]. Aromatic lactic acid derivatives, such as 3‐(3,4‐dihydroxyphenyl)‐2‐hydroxypropanoic acid and 2‐hydroxy‐3‐(4‐hydroxyphenyl) propanoic acid, exhibit antioxidant and anti‐inflammatory properties, inhibiting the production of inflammatory mediators (NO, TNF‐α, and IL‐1β) in mouse microglia or brain tissue, indicating a potential role in modulating neuroimmune responses [39]. The tyrosol derivative 3‐hydroxyphenylethanol also demonstrates neuroprotective and antioxidant effects through free radical scavenging [40]. Additionally, although D‐xylose is primarily used in absorption tests, reports indicate abnormal absorption in MS patients, suggesting potential alterations in gut function or microbial metabolism [41]. The screening rationale and links to previous MS or neuroinflammation studies provide valuable clues for further exploration of neuroimmune regulation; however, the direct effects of most metabolites on MS still require validation through clinical and mechanistic studies.

This study indicates that certain microbial taxa within the G–M–T network may influence the onset and progression of MS through immune regulation and metabolic pathways. For example, taxa within the G–M–T network, including *Acinetobacter*, *Pseudomonas*, *Parabacteroides distasonis*, and *Oscillospira*, have previously been shown to be closely associated with MS [42–45]. Additionally, some proinflammatory bacteria, such as *Bacteroidetes*, *Akkermansia*, and *Ruminococcus*, are often enriched in MS patients [46], with *Akkermansia* and *Acinetobacter* capable of inducing proinflammatory responses, potentially exacerbating autoimmune inflammation via activation of T cells or other immune cells [47]. In contrast, SCFA‐producing bacteria, including *Bifidobacterium*, *Faecalibacterium*, and *Oscillospiraceae*, are typically reduced in MS patients [46, 48, 49]. This decrease in SCFAs can impair Treg differentiation through multiple pathways, thereby weakening immune regulation [50–52]. Furthermore, the major capsular polysaccharide A of *Bacteroides* can mediate the conversion of CD4^+^ T cells into IL‐10‐producing FoxP3^+^ Tregs via toll‐like receptor 2, while simultaneously suppressing Th17 responses [52]. Notably, we also detected these previously reported taxa in our study, along with several newly identified potential MS‐associated microbes, although functional validation experiments have not yet been performed, representing an important direction for future research. In patients with MS, increased proinflammatory bacteria and reduced SCFA‐producing bacteria may disrupt the Treg/Th17 balance and affect inflammatory signaling pathways. Therefore, gut microbial dysbiosis in MS patients may be closely associated with immune dysregulation and altered inflammatory status, potentially contributing to disease onset and progression.

The single‐cell analysis revealed that these DEGs primarily regulate immune cell interactions through the galectin signaling pathway (LGALS9–CD44/CD45). Galectin‐9 binds to CD44, enhancing the stability and function of inducible regulatory T cells [53], and may suppress autoimmune responses and induce immune tolerance by forming a complex with the TGF‐β receptor. Galectin‐9 can also interact with the immune receptor phosphatases CD45/CD148, strengthening their interaction with TIM‐3 [54]. In mouse models, Gal‐9 activates NK cells via CD44 [55]. Galectin‐9 is considered an immune checkpoint molecule with multiple immunoregulatory functions. These findings provide a theoretical basis for the role of the galectin signaling pathway in immune regulation in MS.

In recent years, the microbiota–gut–brain axis has been recognized as playing a critical role in both health and neurological disorders. Studies have shown that gut microbes and their metabolites are closely associated with brain structure, function, and cognitive performance, such as being linked to the pathological features and metabolic pathways of cerebral small vessel disease [56], influencing brain networks related to working memory [57], and correlating with structural and functional brain alterations as well as cognitive and emotional outcomes in Alzheimer’s disease and major depressive disorder [58, 59]. Multimodal neuroimaging studies further indicate that brain functional networks mediate the relationships between gut microbiota and cognition, sleep, and executive function [60, 61]. In MS, our findings support the notion that specific gut microbes and their metabolites may influence disease progression by modulating brain functional networks and neurometabolic pathways. These results not only provide new insights into the pathophysiology of MS but also suggest that targeting gut microbiota may serve as a potential strategy for future adjunctive interventions.

Although our integrative framework provides new insights into the potential regulatory roles of gut microbial metabolites in MS, several limitations should be acknowledged. First, this study is based on publicly available multiomics datasets, and heterogeneity among datasets, including differences in sample sources, sequencing platforms, and analytical pipelines, may introduce bias into the results. Second, as a computational and integrative analysis, the findings primarily rely on in silico predictions, and the lack of direct experimental validation limits causal inference. Future studies incorporating in vitro and in vivo MS‐related models will be necessary to validate the biological relevance of the identified key targets and signaling pathways. In addition, the coverage of the gutMGene database remains limited and may not fully capture the dynamic and context‐dependent nature of gut microbial metabolism. These limitations highlight the need for further experimental and longitudinal studies to confirm and extend our findings.

## 5. Conclusion

This study integrates network pharmacology, ML, and single‐cell transcriptome analysis to systematically investigate the potential regulatory associations between gut microbiota‐derived metabolites and key targets in MS. CASP3 was identified as a core target through multiomics approaches. Gut microbial metabolites, including L‐isoleucine, aromatic lactic acid derivatives, 3‐hydroxyphenethyl alcohol, and D‐xylose, were predicted to interact with these core targets and may be involved in TNF, MAPK, and IL‐17 signaling pathways, thereby potentially influencing neuroimmune responses. In addition, specific microbial taxa within the G–M–T network, such as *Akkermansia*, *Bacteroides*, and *Bifidobacterium*, may contribute to MS pathogenesis through immune regulation and metabolic pathways. Single‐cell analysis further highlighted the crucial role of the galectin signaling pathway in immune cell interactions within CSF. Overall, these findings suggest that gut microbial dysbiosis and its metabolites may contribute to MS‐related neuroimmune dysregulation, providing a theoretical framework for future mechanistic and experimental studies. However, these observations require experimental validation in both in vitro and in vivo MS models to further assess their translational potential.

## Author Contributions

Li Li, Wenbin Xu, and Hongwei Liu were responsible for proposing and designing this study. During the review process, Hongwei Liu, Wenbin Xu, and Zhinan Ye helped with data analysis and resolved any disagreements. The manuscript was written by Li Li, Wenbin Xu, and Hongwei Liu with Zhinan Ye’s assistance, who also supervised the project and provided resources. The final manuscript was shaped by contributions from all the authors, who discussed the results together.

## Funding

No financial assistance was provided for this study.

## Ethics Statement

This study was conducted using publicly available and de‐identified datasets, including the Gene Expression Omnibus (GEO), gutMGene, genome‐wide association study (GWAS) summary statistics, AlphaFold, and PubChem databases, as well as online analytic platforms, such as Similarity Ensemble Approach (SEA) and SwissTargetPrediction (STP). All datasets were anonymized and collected from publicly available repositories. Therefore, no additional institutional review board approval or informed consent was required.

## Consent

The authors have nothing to report.

## Conflicts of Interest

The authors declare no conflicts of interest.

## Data Availability

The data that support the findings of this study are available from the corresponding author upon reasonable request.
